# Dynamics and Sources of Soil Organic C Following Afforestation of Croplands with Poplar in a Semi-Arid Region in Northeast China

**DOI:** 10.1371/journal.pone.0086640

**Published:** 2014-01-23

**Authors:** Ya-Lin Hu, Li-Le Hu, De-Hui Zeng

**Affiliations:** 1 State Key Laboratory of Forest and Soil Ecology, Institute of Applied Ecology, Chinese Academy of Sciences, Shenyang, People’s Republic of China; 2 Chinese Research Academy of Environmental Sciences, Beijing, People’s Republic of China; DOE Pacific Northwest National Laboratory, United States of America

## Abstract

Afforestation of former croplands has been proposed as a promising way to mitigate rising atmospheric CO_2_ concentration in view of the commitment to the Kyoto Protocol. Central to this C sequestration is the dynamics of soil organic C (SOC) storage and stability with the development of afforested plantations. Our previous study showed that SOC storage was not changed after afforestation except for the 0–10 cm layer in a semi-arid region of Keerqin Sandy Lands, northeast China. In this study, soil organic C was further separated into light and heavy fractions using the density fractionation method, and their organic C concentration and ^13^C signature were analyzed to investigate the turnover of old vs. new SOC in the afforested soils. Surface layer (0–10 cm) soil samples were collected from 14 paired plots of poplar (*Populus* × *xiaozhuanica* W. Y. Hsu & Liang) plantations with different stand basal areas (the sum of the cross-sectional area of all live trees in a stand), ranging from 0.2 to 32.6 m^2^ ha^−1^, and reference maize (*Zea mays* L.) croplands at the same sites as our previous study. Soil *Δ_C_* stocks (*Δ_C_* refers to the difference in SOC content between a poplar plantation and the paired cropland) in bulk soil and light fraction were positively correlated with stand basal area (*R* ^2^ = 0.48, *p*<0.01 and *R* ^2^ = 0.40, *p* = 0.02, respectively), but not for the heavy fraction. SOC_crop_ (SOC derived from crops) contents in the light and heavy fractions in poplar plantations were significantly lower as compared with SOC contents in croplands, but tree-derived C in bulk soil, light and heavy fraction pools increased gradually with increasing stand basal area after afforestation. Our study indicated that cropland afforestation could sequester new C derived from trees into surface mineral soil, but did not enhance the stability of SOC due to a fast turnover of SOC in this semi-arid region.

## Introduction

Afforestation of degraded croplands has occurred globally within the framework of the Kyoto Protocol [1], and has the potential to mitigate the rising atmospheric CO_2_ concentration caused by anthropogenic emissions [2], [3]. Consequently, changes of soil organic C (SOC) stocks after cropland afforestation have been well studied [3]–[8], considering the fact that soil has the greatest potential C sink capacity and the longest time period [9]–[11]. However, most previous studies have suggested that there were large variations in the direction and magnitude of SOC stock changes following afforestation of croplands, which are related to previous land-use, climate, soil texture, tree species, stand age, and management practices [3], [4], [8]. In addition, the changes of SOC stocks are often not detected by conventional methodologies within a short-time frame for most experiments due to the small changes in soil C when compared with the size of SOC reservoir [6], [9]. In order to accurately assess small changes in SOC stocks and stability following cropland afforestation, it is necessary to investigate the dynamics in different SOC pools characterized by different physical and chemical properties, microbial degradability and turnover time [9].

Soil organic matter (SOM) can be divided into discrete fractions with different stability and ecological functions [12], [13]. Density fractionation physically separates SOC into light fraction and heavy fraction (LF and HF, respectively), which has been increasingly used to assess SOC dynamics induced by land use change and management practices [8], [14]–[17]. However, soil fractionation and C analysis only provide information on net changes of SOC, but not for soil C balance between the loss of old C and the input of new C. The ^13^C natural abundance technique offers an elegant approach to quantify the relative contribution of new vs. old SOC, for example, where C_3_ plants (δ^13^C ca. −28‰) grow on soils derived from C_4_ crops (δ^13^C ca. −12‰) [9]. Several studies have elucidated the dynamics in SOC following cropland afforestation using soil fractionation techniques combined with the stable C isotope techniques [9], [18]–[20], but this approach has not been used in semi-arid regions.

The SOC stocks are determined by the balance between the inputs of C derived from litterfall and rhizodeposition and the losses of C mainly through soil organic matter decomposition [1], [21]. Therefore, the dynamics of SOC stocks following afforestation are not only correlated with stand age, but also with tree density that influences soil microclimatic conditions and the amount of litterfall [1], [22], [23]. Stand basal area (BA, the sum of the cross-sectional area of all live trees in a stand) can integrates information of both stand age and tree density, which is more feasible to evaluate the dynamics and sources of SOC following afforestation considering the important roles of trees in SOC inputs from litter and root exudates.

Understanding the changes in SOC stocks and stability following afforestation in semi-arid regions is important principally because of the vast area involved with 2.4 billion ha or ∼17.7% of total global land surface area [24], and the different changes in soil C stocks and stability after afforestation compared with humid regions [25], [26]. Poplar (*Populus*) species is one of the most widely grown trees on croplands in the Keerqin Sandy Lands, a semi-arid region in northeastern China, in two large scale afforestation programs i.e., the Three-North Shelter Forest Program and the Grain for Green Project. The net changes in above- and below-ground C stocks after afforestation have been well investigated in the Keerqin Sandy Lands [27]–[29]. In this present study, the objectives are to further evaluate the dynamics and sources of SOC in different soil C pools along BA following afforestation of croplands with poplars in this semi-arid region. We hypothesized that: (1) SOC stocks in bulk soil and light fraction would increase following cropland afforestation due to the enhanced inputs of litterfall with increasing BA, and (2) the sources of SOC would gradually convert from crop-derived sources to tree-derived sources because of the decomposition of old soil C and the accumulation of new soil C. To test these hypotheses, we selected 14 paired stands of poplar plantations with different BAs afforested on croplands and adjacent maize (*Zea mays* L.) croplands as controls in the southeastern region of Keerqin Sandy Lands, and then analyzed SOC concentrations and *δ* ^13^C values in bulk soil, LF and HF.

## Materials and Methods

### 2.1 Ethics Statement

This study was carried out on collective-owned lands, and the owners of the lands gave us permission to conduct the study on these stands. The field studies did not involve endangered or protected species.

### 2.2 Site Description and Experimental Design

This study was carried out in the southeastern region of the Keerqin Sandy Lands (42°30′–42°55′N, 122°19′–122°30′E), a typical semi-arid region in northeast China. The climate is temperate continental monsoon. Mean annual temperature is about 5.7°C, ranging from −23.2°C in January to 32.4°C in July (1954–2004). Mean annual precipitation is about 450 mm (ranging from 224 mm to 661 mm during 1954–2004), with more than 60% occurring from June to August, and mean annual potential evaporation ranges from 1300 to 1800 mm with an average length of frost-free season of about 150 days. The soil is a sandy soil with 90.9% sand, 5.0% silt and 4.1% clay, and classified into the Entisol order, Semiaripsamment group (according to the United States Soil Classification System) and developed from sandy parent material through the action of wind [30]. Before croplands are established in this region, the dominant species of the native vegetation include *Agropyron cristatum*, *Arundinella hirta*, *Cleistogenes chinensis*, *Lespedeza davurica* and *Artemisia capillaris* var. *simplex* [31].

Since 1978, a large area of marginal croplands has been afforested with trees in this region under the Three-North Shelter Forest Program and the Grain for Green Project, in order to control windy erosion and desertification. By now, the wind erosion is effectively reduced. In June 2011, we selected 14 poplar (*Populus* × *xiaozhuanica* W. Y. Hsu & Liang, a hybrid of *P. nigra* var. *italica* and *P. simonii*) plantations (ranging from 2 to 20 years old) afforested on maize croplands and 14 adjacent maize cropland stands as control in Kezuohouqi and Zhangwu counties on the basis of a paired-plot experimental design (Table 1). Most of the paired cropland and plantation stands are conterminous except for several cases, and the distance of sampling point in each paired poplar plantation and cropland is less than 500 m. The topography and soil conditions are similar in each paired cropland and poplar plantation, and the slope of each stand is very gentle and less than 5°. All the poplar plantation stands were planted on maize croplands that had cultivated for at least 20 years before afforestation, and the paired croplands were continually planted to maize. Usually, croplands are fertilized with urea fertilizer each year, while fertilizer is no longer used after afforestation.

**Table 1:** Stand location and characteristics.

Polar plantation stand							Cropland stand		
Plot	Location	Elevation(m)	Treeheight (m)	DBH†(cm)	Density(Trees ha^−1^)	Stand basalarea (m^2^ ha^−1^)	Plot	Location	Elevation (m)
F1	42°54′11″N, 122°23′30″E	252	3.35	2.92	1100	0.85	C1‡	42°54′04″N, 122°23′32″E	255
F2	42°37′06″N, 122°20′54″E	166	4.38	4.62	1075	1.85	C2	42°37′04″N, 122°20′52″E	166
F3	42°54′00″N, 122°23′25″E	252	4.80	6.35	850	2.92	C3	42°53′59″N, 122°23′23″E	251
F4	42°53′04″N, 122°24′08″E	250	5.26	5.73	1275	3.65	C4	42°52′58″N, 122°24′09″E	250
F5	42°54′02″N, 122°25′32″E	247	6.85	7.39	1300	6.11	C5	42°54′00″N, 122°25′34″E	246
F6	42°53′18″N, 122°24′11″E	251	8.77	9.21	1100	8.20	C6	42°53′17″N, 122°24′07″E	252
F7	42°59′25″N, 122°20′25″E	244	8.95	11.58	825	9.24	C7	42°59′26″N, 122°20′20″E	244
F8	42°37′58″N, 122°22′24″E	186	15.80	13.37	750	10.69	C8	42°38′01″N, 122°22′24″E	185
F9	42°54′13″N, 122°23′36″E	252	15.20	15.10	700	13.15	C9	42°54′17″N, 122°23′41″E	251
F10	42°53′59″N, 122°25′10″E	249	18.70	10.54	1575	14.62	C10	42°53′56″N, 122°25′07″E	248
F11	42°55′42″N, 122°24′39″E	246	17.76	13.16	1025	15.69	C11	42°55′42″N, 122°24′37″E	246
F12	42°37′06″N, 122°20′58″E	165	30.72	22.00	625	24.79	C12	42°37′04″N, 122°20′52″E	165
F13	42°54′25″N, 122°23′28″E	250	20.68	17.11	1150	28.51	C13	42°54′23″N, 122°23′28″E	250
F14	42°37′05″N, 122°21′06″E	164	29.87	22.90	775	32.60	C14	42°37′06″N, 122°21′05″E	165

† DBH: Diameter at breast height (1.3 m); an average value for all living trees in each plot.

‡ All croplands were planted to maize.

### 2.3 Stand Investigation and Soil Sampling

One 20 × 20 m plot was established in each stand. For poplar plantations, the diameter at breast height (DBH) and tree height were measured for all live trees in each plot. DBH was measured at breast level (1.3 m above ground) using a caliper. For measurement of tree height, we used a long pole to extend vertically to the top of the tree and then measured the length of the pole. The *BA* (m^2^ ha^−1^) was calculated from measurements of the *DBH* (cm) of all trees in a known area (*A,* ha), which was expressed as:
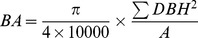
(1)


Considering the changes of soil organic C stocks were only observed in 0–10 cm layer in our previous study [28] at the same sites, soil samples in the surface layer were only collected in this present study. Four soil cores were sampled randomly using an auger (2.5 cm in diameter) at the surface mineral soil layers (0–10 cm), and thoroughly mixed to form a homogenized sample for each stand (i.e., a total of 28 samples including 14 samples from poplar plantations and 14 samples from croplands). Soil samples were dried at room temperature (20 °C) and then passed through a mesh sieve with a size of 2 mm. Soil bulk density (*ρ*) was determined at three randomized sampling points in each plot for calculation of SOC content. For measurement of soil bulk density, a metal corer (volume is 100 cm^3^) was driven into the soil at the desired depth, and then soil samples were oven dried at 115°C for 24 h and weighed. Soil bulk density was calculated as:
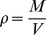
(2)where *M* is dry mass of soil and *V* is volume of soil (i.e., 100 cm^3^).

### 2.4 Soil Density Fractionation

A soil sample was physically separated into two pools by the modified density fractionation method of Sohi et al. [32]. Briefly, 10 g of air-dried soil (<2 mm) were placed in a centrifuge tube with 40 mL sodium iodide (NaI) solution at a density of 1.7 g cm^−3^. The tubes were shaken up and down by hand for ten times. The release of light fraction was accelerated by sonication at 58 Watts for 180 s using a sonicator (Bilon96, Bilon Instruments Co., Ltd, China). After sonication was finished, the tubes were centrifuged at 6000 rpm for 20 min. The floating material was aspirated together with the NaI solution from the surface of tubes, and then filtered using Whatman GF/A filter papers. This procedure was repeated three times. The material collected on the filter paper (light fraction, LF) and the residue remaining in the centrifuge tube (heavy fraction, HF) were rinsed thoroughly with deionized water and collected. The samples of LF and HF were dried at 60°C for 48 h.

### 2.5 SOC Concentration and ^13^C Analysis

The samples of bulk soil, LF and HF were ground to a fine powder with a ball mill and analyzed for SOC concentration and *δ* ^13^C. SOC concentration was determined using the Walkey and Black K_2_Cr_2_O_7_–H_2_SO_4_ oxidation method [33]. The isotope ratio ^13^C/^12^C was determined using isotope ratio mass spectrometer (Finnigan DELTAplusXP, Thermo Fisher Scientific, USA), and the ^13^C abundance was expressed in delta-units (*δ* ^13^C, ‰) according to the following equation:
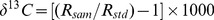
(3)where *R*_sam_ is the ^13^C/^12^C ratio of soil sample and *R*_std_ is the ^13^C/^12^C ratio of the international Pee Dee formation belemnite carbonate standard (PDB).

### 2.6 Data Calculation and Statistical Analysis

Soil organic C content of bulk soil, LF and HF was calculated as:
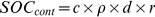
(4)where *SOC*_cont_ was soil organic C content of bulk soil, LF or HF; *c* was SOC concentration of bulk soil, LF or HF; *ρ* was soil bulk density; *d* was soil depth (i.e. 10 cm); and *r* was the dry mass ratio of LF or HF to bulk soil.

We estimated the sources of SOC in the poplar plantations based on an isotope mass balance and *δ* ^13^C values, and the fractional tree-derived SOC (*F*_tree-C_) was calculated using a two-component mixing equation [34]:
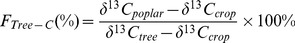
(5)where *δ^13^C*_poplar_ and *δ^13^C*_crop_ values are actual measured values in bulk soil, LF or HF in poplar plantation and its paired cropland, respectively. The *δ^13^C*_tree_ value is the measured *δ* ^13^C of poplar leaf litter (−29.63‰). Subsequently, the mass of tree-derived SOC (SOC_tree_) and crop-derived SOC (SOC_crop_) were calculated as:



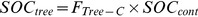
(6)

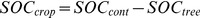
(7)


All statistical analyses were done using the open source statistical software R version 2.14.1. Paired *t* tests were used to examine the changes of SOC concentration and SOC content in bulk soil, LF and HF between poplar plantations and the paired croplands. To test the dynamics and sources of SOC after afforestation, the relationships of BA and SOC content, and BA and *δ* ^13^C of bulk soil, LF and HF in poplar plantation stands were tested with a linear regression analysis. The significance level was set at *α = *0.05 for all the statistical analyses unless otherwise noted.

## Results

### 3.1 SOC in Bulk Soil, Light Fraction and Heavy Fraction

**Figure 1:**
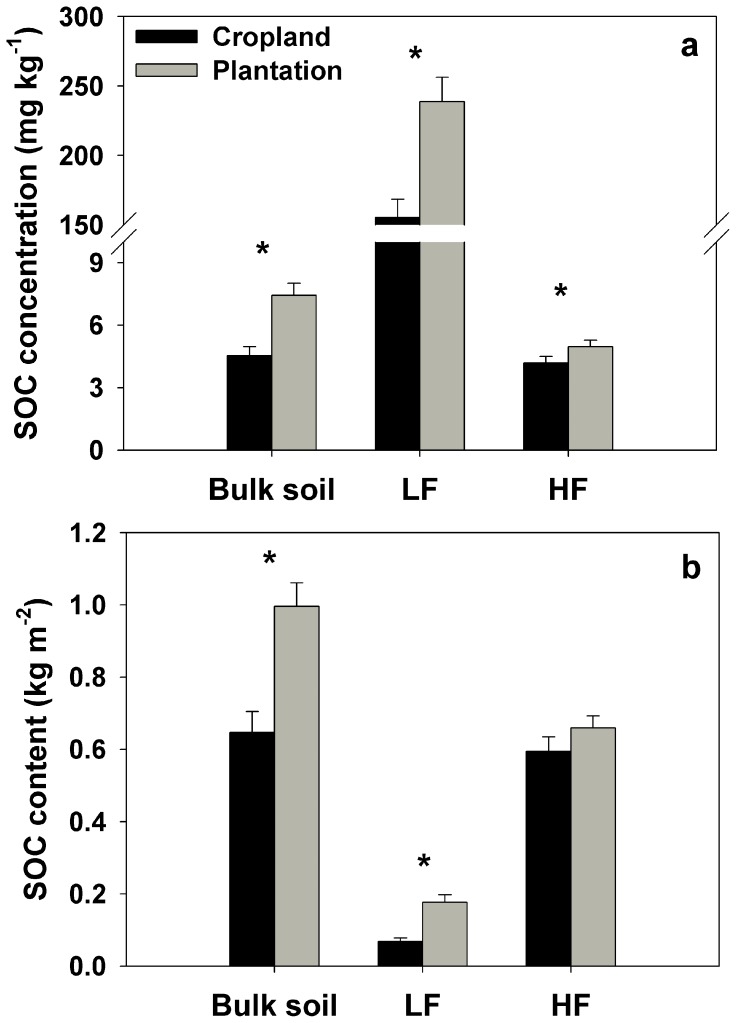
Averages of SOC concentration (a) and content (b) of bulk soil, LF and HF in croplands and poplar plantations. The asterisk above the bars indicates a significant difference between cropland and poplar plantation at *α = *0.05 level. The vertical error bars are standard errors of the means (*n* = 14).

Across all popular plantation stands, the average SOC concentration and SOC content in bulk soil were 64% and 54% higher than that across croplands, respectively (all *p*<0.001) (Fig. 1). SOC concentrations in bulk soil ranged from 4.67 to 12.50 g kg^−1^ in poplar plantations, and from 2.39 to 7.28 g kg^−1^ in croplands. SOC contents had a range from 0.67 to 1.54 kg C m^−2^ in poplar plantations, and a range from 0.38 to 1.06 kg C m^−2^ in cropland stands.

SOC concentrations in LF and HF in poplar plantations (an average of 238 g kg^−1^ in LF and 4.97 g kg^−1^ in HF, respectively) were also significantly higher than that in croplands (an average of 155 g kg^−1^ in LF and 4.19 g kg^−1^ in HF, respectively) (Fig. 1a). SOC was mainly stored in the HF pools in both poplar plantations and croplands. Average values of SOC content in LF and HF were 0.18 and 0.66 kg m^−2^ in poplar plantations, and 0.07 and 0.59 kg m^−2^ in croplands, respectively. SOC content in the LF of poplar plantations was significantly higher than that in croplands (*p*<0.001), but not for HF (*p* = 0.12) (Fig. 1b).

### 3.2 Changes of SOC in Bulk Soil, LF and HF with BA

**Figure 2:**
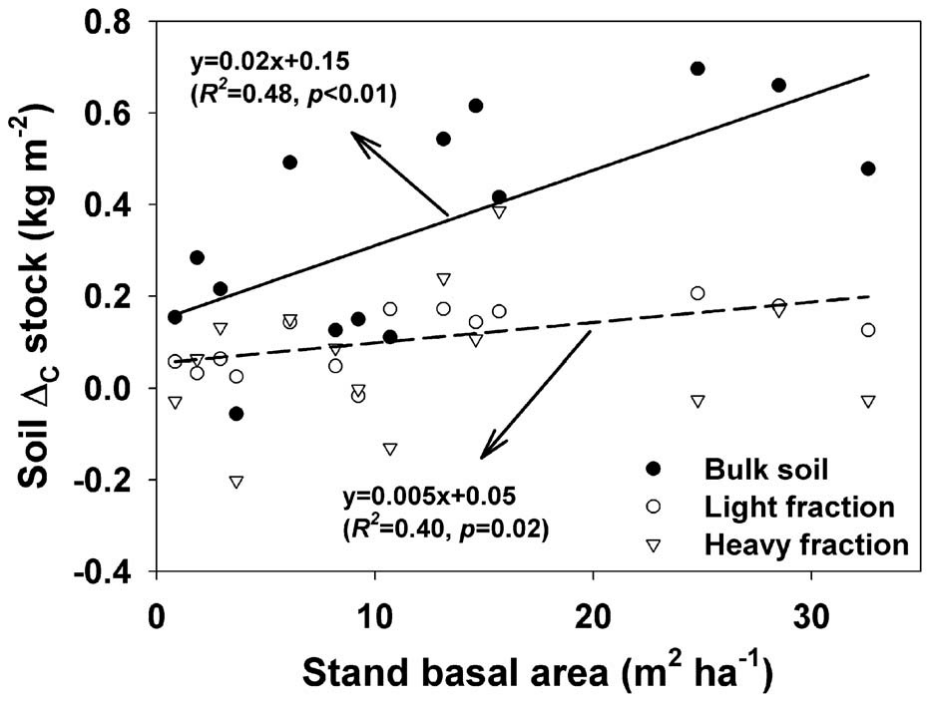
Dynamics of soil *Δ_C_* stocks in bulk soil, LF and HF along stand basal area in poplar plantation stands. Solid line is regression for the bulk soil, and dash line is regression for light fraction.

Soil *Δ_C_* stocks (*Δ_C_* refers to the difference in SOC content between a poplar plantation and the paired cropland) in bulk soil had a linear increase trend with increasing BA (Fig. 2). Similarly, there was a significant positive correlation between soil *Δ_C_* stocks in LF and BA, but not for HF.

### 3.3 Soil δ^13^C in Poplar Plantations

**Figure 3:**
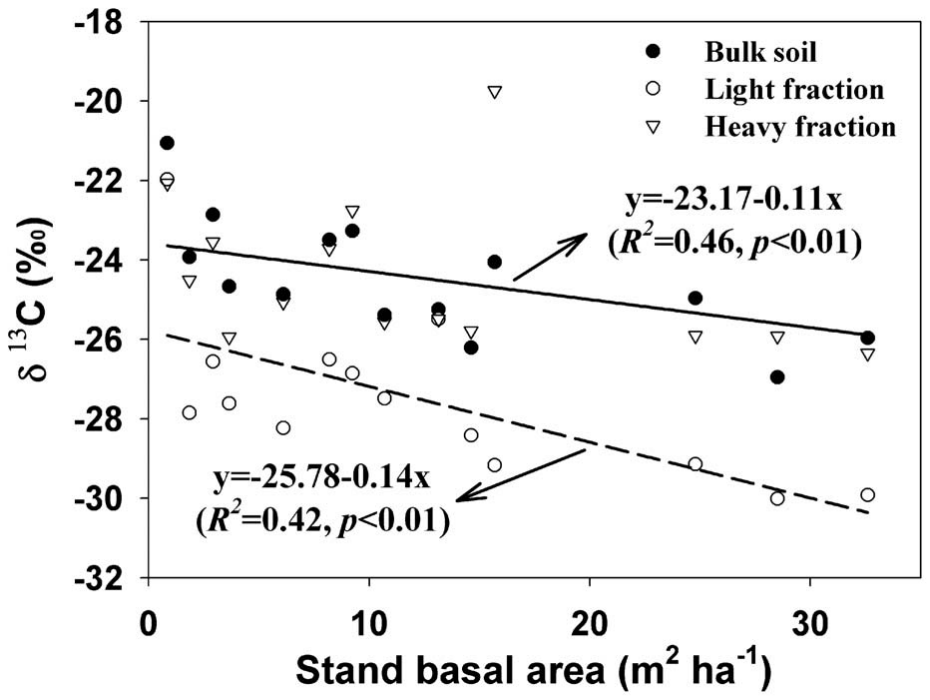
Dynamics of soil *δ* ^13^C in bulk soil, LF and HF along stand basal area in poplar plantations. Solid line is regression for bulk soil, and dash line is regression for light fraction.

Soil *δ* ^13^C values in bulk soil and LF were negatively correlated with BA (Fig. 3). Soil *δ* ^13^C in bulk soil was depleted from −21‰ (BA was 0.85 m^2^ ha^−1^) to −27‰ (BA was 28.51 m^2^ ha^−1^), and soil *δ* ^13^C in LF was depleted from −22‰ (BA was 0.85 m^2^ ha^−1^) to −30‰ (BA was 28.51 m^2^ ha^−1^). However, the relationship between soil *δ* ^13^C in HF and BA was not significant (*p* = 0.18). Soil *δ* ^13^C in HF (average value of −24‰) were significantly higher than that in LF (an average value of −28‰) in poplar plantations (*p*<0.001).

**Figure 4:**
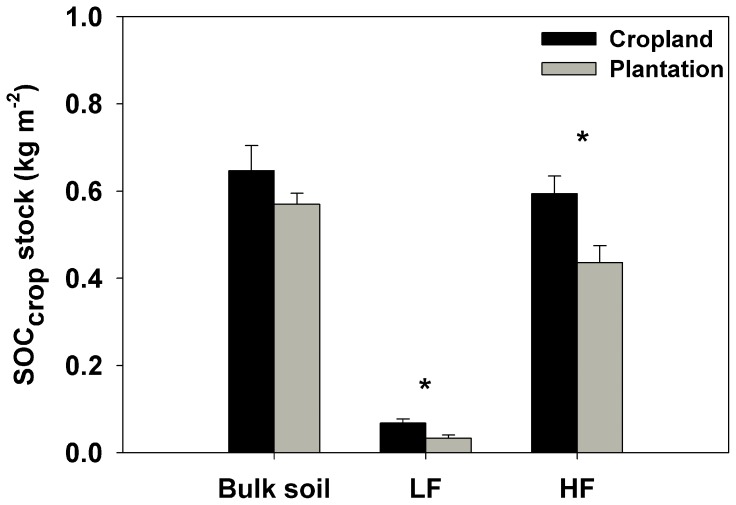
Averages of SOC_crop_ contents in bulk soil, LF and HF in croplands and poplar plantations. The asterisk above the vertical bars indicates a significant difference between cropland and poplar plantation at *α = *0.05 level. The vertical error bars are standard errors of the means (*n* = 14).

**Figure 5:**
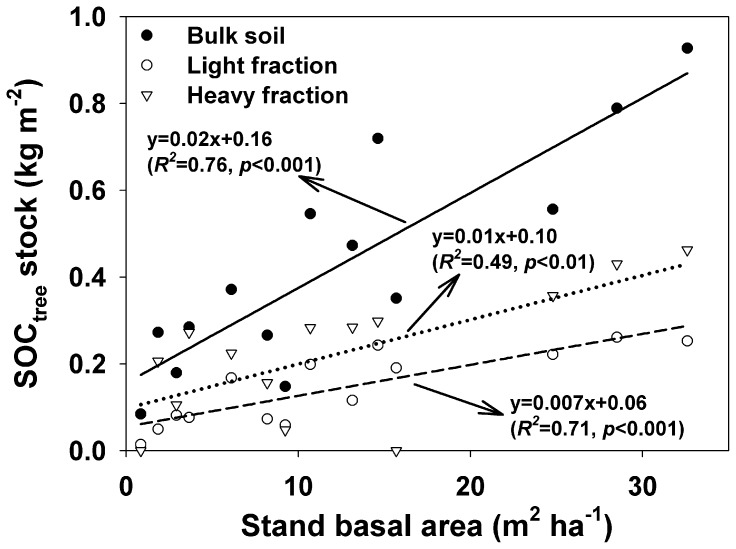
Dynamics of SOC_tree_ contents in bulk soil, LF and HF along stand basal area in poplar plantations. Solid line is regression for bulk soil, and dash line is regression for light fraction, and dot line is regression for heavy fraction.

SOC_crop_ (SOC derived from crops) content in bulk soil of poplar plantations was slightly lower as compared with SOC content in croplands (*p* = 0.17), while SOC_crop_ contents in LF and HF were 51% and 27% lower, respectively (all *p*<0.05) (Fig. 4). SOC_tree_ (SOC derived from poplar trees) contents in bulk soil, LF and HF were all increased significantly with increasing BA (Fig. 5). SOC_tree_ contents in bulk soil ranged from 0.08 kg m^−2^ in the poplar stand (BA was 0.85 m^2^ ha^−1^) to 0.93 kg m^−2^ in the poplar stand (BA was 32.6 m^2^ ha^−1^). The percentages of SOC_tree_ to total SOC content in poplar plantations were on average 40% in bulk soil, 77% in LF, and 33% in HF.

## Discussion

### 4.1 Changes of SOC in Bulk Soil, LF and HF

In this study, the afforested soil had higher SOC content than croplands (an average of 0.35 kg C m^−2^) in the surface soils, implying that afforestation with hybrid poplar on croplands could sequester 12.8 Mg CO_2_ ha^−1^ into surface mineral soils in this semi-arid region. Similarly, Mao et al. [29] observed that SOC sequestration in 1 m depth was 1.9 kg C m^−2^ in a 20-year-old poplar plantation that was afforested on the marginal agricultural land. Li et al. [35] reported that soil sequestered 0.39 kg C m^−2^ in 0–15 cm depth in a 25-year-old Mongolian pine (*Pinus sylvestris* var. *mongolica*) afforested on active sand dune in the Keerqin Sandy Lands. Increased SOC stocks induced by afforestation with poplar trees on croplands were also reported in other regions [36]–[38]. When land use change from crop to forest, an average increase of 18% in SOC stock was observed, and afforested broadleaved tree species were more effective in sequestering CO_2_ into soils [3].

Furthermore, this present study showed that soil *Δ_C_* stocks increased with increasing BA, suggesting the potential capacity of storing soil C would enhance with the increase of BA after afforestation. It is consistent with results in the literature: for example, Vesterdal et al. [39] found that SOC contents in 0–5 cm increased with stand age after afforestation with Norway spruce (*Picea abies* (L.) Karst) on former arable lands. Sartori et al. [38] also observeed that SOC stocks in 0–10 cm layer had an increasing trend along a choronosequence of poplar plantations in the Columbia Plateau, Oregon, USA. Mao et al. [29] and Arevalo et al. [36] all found that SOC stocks increased with stand age though there was a loss of SOC in the early stage after poplar plantations were established on croplands. It has been proposed that the increases of soil C inputs from litterfall with increasing stand age or tree density, and the lack of tillage disturbance cause the increase in soil C stocks after cropland afforestation [8], [19].

The light fraction of SOM is commonly referred to as plant-like SOM with high C concentration, and the heavy fraction contains more decomposed SOM with lower C concentration [12]. Consistently, we found that SOC concentrations (ranging from 99 to 270 g kg^−1^ in croplands and 138 to 351 g kg^−1^ in poplar plantations, respectively) in the LF were obviously higher than in the HF (ranging from 2.72 to 5.89 g kg^−1^ in croplands and 2.89 to 7.01 g kg^−1^ in poplar plantations, respectively) in this present study. However, SOC content in the HF accounted for 89% of SOC in croplands and 79% of SOC in poplar plantations (Fig. 1b), indicating that SOC stocks were mainly distributed in the HF in both croplands and poplar plantations. In general, SOC contents in the LF accounted for 17–47% of total SOC contents in the surface mineral soil in a temperate zone [12], and the larger mass of the heavy fraction in soils was also observed by the other studies [8], [15]–[17], [40].

Changes of SOC stock in LF are usually more sensitive to land use change and management practices [14]–[17]. In this study, we observed that SOC concentration and content in the LF were all significantly increased after afforestation (Fig. 1). Similarly, the increases of SOC concentration in the LF following cropland afforestation were also observed by Laik et al. [41]. Furthermore, Li et al. [35] found that afforestation with Mongolian pine on active sand dunes resulted in an increase of light fraction C concentration in Keerqin Sandy Lands. There was a significant positive relationship between soil *Δ_C_* stocks in LF and BA, indicating the gradual increases of SOC sequestration into the LF with increasing BA. It is consistent with the results of Marin-Spiotta et al. [42] who found that the mass and SOC concentrations in the LF increased along a chronosequence of natural reforestation of abandoned tropical pastures. The increases of soil C in the LF could be associated with the enhanced C input with increasing BA [14], considering the significant positive relationship between SOC_tree_ in the LF and BA (Fig. 5).

Soil organic C content in the HF plays an important role in the stability of SOC associated with its slow decomposition due to physical protection. However, we did not observe the increase of SOC stock in HF after afforestation in this semi-arid region. It implies that SOC stability might not be enhanced after afforestation with poplar on croplands. Huang et al. [14] also reported that there were no significant changes in SOC stock in the heavy fraction after afforestation on grasslands. However, Clark et al. [18] observed that the stability of SOC increased when native forests were allowed to invade abandoned agricultural fields in western New England. SOC stability is controlled by soil texture rather than land use management [5]. The enhancement of SOC stability after afforestation more likely occurred in soils with more clay and silt, and under climate conditions with more precipitation and warmer temperature [4], [18], [26].

### 4.2 Turnover of SOC

The SOC stocks are determined by the balance between the input of C derived from plant litter and the loss of C mainly through soil organic matter decomposition [1], [21]. Our results showed that SOC derived from crops (i.e., old SOC) in the LF and HF in poplar plantations was all significantly lower than that in croplands, though the difference of SOC_crop_ in bulk soil was not significant (Fig. 4), implying that cropland afforestation led to the loss of old SOC. However, the new SOC derived from poplar trees in bulk soil, LF and HF all gradually increased with increasing BA (Fig. 5). Consistently, a net loss of old SOC and a gain of new SOC after cropland or grass afforestation were also observed in several other studies [14], [19], [20]. Our results imply that the increased C inputs from trees (litter and roots) following afforestation are the major causes enhancing soil C sequestration rather than the inhibition of soil old C decomposition.

Though there was a positive relationship between SOC_tree_ in the HF of the poplar plantations and BA, we could not infer that the long-term stability of SOC derived from trees would be enhanced considering the fast turnover of SOC_crop_ in the HF in poplar plantation stands in this semi-arid region. Galdo et al. [9] and Dondini et al. [19] suggested that afforestation could enhance SOC stability because they did not find the loss of old soil SOC due to the formation of soil aggregates. Furthermore, Paul et al. [5] suggested that the presence of soil C stabilization processes did not necessarily mean that recently incorporated soil C will also be effectively stabilized. In our study, a fast replacement of the old SOC by new SOC might be associated with the weak physical protection in the sandy soil [13], and the frequent drying-rewetting in the semi-arid region [43].

## Conclusions

Our results confirm that cropland afforestation with poplars has the potential to sequester C rapidly into soils considering the gradual increases of SOC following afforestation because of the substantial replenishment of old soil C derived from crops by new C derived from trees. However, soil C sequestration was mainly caused by the increase in soil LF, but no significant changes of SOC content were observed in soil passive C pool due to a loss of old soil C in HF in this semi-arid region. It implies that the stability of soil organic C is not enhanced after a short-term afforestation on croplands.
